# Development of Open-Angle Glaucoma in Adults With Seropositive Rheumatoid Arthritis in Korea

**DOI:** 10.1001/jamanetworkopen.2022.3345

**Published:** 2022-03-21

**Authors:** Seung Hoon Kim, Sung Hoon Jeong, Hyunkyu Kim, Eun-Cheol Park, Suk-Yong Jang

**Affiliations:** 1 Department of Preventive Medicine, Yonsei University College of Medicine, Seoul, Republic of Korea; 2 Institute of Health Services Research, Yonsei University, Seoul, Republic of Korea; 3 Department of Public Health, Yonsei University Graduate School, Seoul, Republic of Korea; 4 Department of Psychiatry, Yonsei University College of Medicine, Seoul, Republic of Korea; 5 Department of Healthcare Management, Graduate School of Public Health, Yonsei University, Seoul, Republic of Korea

## Abstract

**Importance:**

Although evidence is emerging that autoimmunity may be associated with neurodegeneration in glaucoma (beyond intraocular pressure-mediated damage), there is limited evidence connecting rheumatoid arthritis (RA), the most common autoimmune disease, with the risk of developing primary open-angle glaucoma (POAG).

**Objective:**

To investigate whether RA is associated with increased risk of POAG among Korean older adults.

**Design, Setting, and Participants:**

A nationwide propensity-matched cohort study was conducted using data from the Korean National Health Insurance Service-Senior cohort from 2002 to 2013. Data analysis was performed from November 2020 to July 2021.

**Exposures:**

New onset RA.

**Main Outcomes and Measures:**

The main outcome was development of POAG. The Kaplan-Meier method was used to calculate the cumulative incidence of POAG, and the incidence rate of POAG was estimated using a Poisson regression. A Cox proportional hazards regression model was used to investigate associations between RA and risk of POAG.

**Results:**

Among 10 245 participants, 7490 (73.1%) were women, and the mean (SD) age was 67.70 (4.84) years. A total of 2049 patients with incident seropositive RA and 8196 time-dependent, propensity score–matched, risk-set controls were included. POAG developed in 86 of 2049 patients with RA and 254 of 8196 matched controls. The cumulative incidence of POAG was higher in the RA cohort than in the matched controls. In the RA cohort, the incidence rate of POAG was 981.8 cases per 100 000 person years (95% CI, 794.3-1213.7 cases per 100 000 person years), whereas in the matched controls, the incidence rate was 679.5 cases per 100 000 person years (95% CI, 600.8-768.3 cases per 100 000 person years). Patients with RA were more likely to develop POAG than the matched controls (hazard ratio [HR], 1.44; 95% CI, 1.13-1.84). Increased POAG risk in the RA cohort was predominantly observed 2 years into the follow-up period (HR, 1.83; 95% CI, 1.28-2.61) and in those aged 75 years or older (HR, 2.12; 95% CI, 1.34-3.35).

**Conclusions and Relevance:**

These findings suggest that RA is associated with a higher risk of developing POAG, especially within 2 years after diagnosis or among patients aged 75 years or older. There may be a common pathophysiological pathway between RA and POAG that is possibly immune mediated, and the nature of this association warrants further investigation.

## Key Points

**Question:**

Do adults with seropositive rheumatoid arthritis develop primary open-angle glaucoma more often than those without rheumatoid arthritis?

**Findings:**

In this cohort study of 10 245 older Korean adults risk-set matched using propensity score, patients with rheumatoid arthritis were more likely than matched controls to subsequently develop primary open-angle glaucoma, with hazard ratios ranging from 1.44 to 2.12.

**Meaning:**

These findings suggest that rheumatoid arthritis is associated with subsequent development of glaucoma; the possibility of an immune-mediated common pathophysiological pathway warrants further investigation.

## Teaser

This cohort study examines whether seropositive rheumatoid arthritis is associated with an increased risk of subsequently developing primary open-angle glaucoma among adults in Korea.

## Introduction

Rheumatoid arthritis (RA) is a progressive, inflammatory, autoimmune disease characterized by articular and extra-articular involvement.^1^ Several factors, including autoantibodies, immune complexes, T cell–mediated antigen-specific responses, and T cell–independent cytokine networks, are involved in the pathogenesis of RA.^2^

Glaucoma is a leading cause of irreversible blindness,^3,4^ and older patients with glaucoma are more likely to have visual impairment,^5^ visual field defects increasing the likelihood of falls,^6,7^ and visual field progression associated with cognitive decline.^8^ Thus, finding pathogenic components for glaucoma in older adults is very important from a clinical and public health perspective.

Well-known factors associated with increased risk of primary open-angle glaucoma (POAG),^9^ the most common subtype of glaucoma,^10^ include older age and family history, but the most important factor is increased intraocular pressure (IOP).^11^ Primary management of POAG is lowering the IOP to a target level at which the clinician believes the disease progression will slow sufficiently.^12^ However, in many patients, optic nerve cupping and visual field damage persist even when the IOP decreases to the below-normal range.^13^ Therefore, various pathogenic mechanisms, such as nitric oxide–based damage and immune-induced neurodestruction, have been proposed as causes of neurological injury in patients with POAG.^14^

Among the several proposed mechanisms of POAG, evidence of immune component involvement in development and progression of POAG is gathering. Autoantibodies and CD4^+^ T cells involved in the pathogenesis of RA are observed in patients with POAG,^15,16^ and enhanced expression of heat shock protein (HSP) is also commonly observed.^3^ Furthermore, etanercept, a tumor necrosis factor–α inhibitor used to control RA progression, reduced retinal ganglion cell (RGC) loss by approximately 50% in a rat glaucoma model.^17^ Given that the role of autoimmunity as a cause of various neurodegenerative diseases has been elucidated,^18^ the possibility of autoimmunity causing glaucoma cannot be ignored.^19^

When considered collectively, it is reasonable to suspect that RA and POAG share common pathogenic pathways, including autoimmune components. Nevertheless, to our knowledge, the association between RA and the risk of developing POAG has not been examined in a nationwide cohort study. Therefore, we aimed to investigate the risk of developing POAG after RA diagnosis in older adults using a nationally representative sample from the South Korean National Health Insurance Service (NHIS)–Senior cohort 2002 to 2013.

## Methods

### Data and Sample

We used data from the 2002 to 2013 NHIS-Senior cohort provided by the NHIS of South Korea. The NHIS is a social security system managed by the government that provides universal health care coverage to Korean citizens. All citizens except those eligible for medical aid are obligated to enroll. The National Health Information Database was developed by NHIS and contains personal information and demographic details of all enrolled.^20^

The NHIS-Senior cohort includes data from 558 147 adults aged 60 years and older, representing a 10% random sample of the 5.5 million people in this age group in the National Health Information Database.^21^ We examined the cohort data retrospectively from 2002 until 2013 for all patients, except those who lost national health insurance eligibility owing to death or emigration, according to the National Health Insurance Act.^21^

This study adhered to the tenets of the Declaration of Helsinki^22^ and was approved by the institutional review board of Severance Hospital at Yonsei University College of Medicine. Informed consent requirement was waived by the institutional review board, as the data did not contain any personally identifiable information. This study followed the Strengthening the Reporting of Observational Studies in Epidemiology (STROBE) reporting guideline.^23^

### Incident RA Cohort

We constructed the incident seropositive RA cohort as follows: First, we included individuals with diseases diagnosed under the M05 code of the *International Statistical Classification of Diseases and Related Health Problems, Tenth Revision (ICD-10)* during outpatient treatment. Next, patients with *ICD-10* code M05 who were prescribed biological agents or any disease-modifying antirheumatic drug (DMARD) were classified,^24,25,26^ including drugs in recently published DMARD literature.^27^ False-positives were reduced by excluding patients who received DMARD prescriptions only once.^28^ Patients classified as seropositive for RA from January 1, 2002, to December 31, 2003, were excluded so that only patients with new diagnoses of RA were enumerated.^28^ Therefore, only individuals who satisfied both diagnostic code M05 and the twice or more DMARD prescription requirement after January 1, 2004 were defined as the incident RA cohort. Finally, we excluded patients who developed POAG before the onset of RA.

### Identification of POAG

To identify only patients with new diagnoses of POAG, excluding glaucoma caused by inflammation owing to RA (eg, uveitis), we defined participants who met the following criteria as newly developed POAG cases referring to other published data: (1) diagnoses of POAG (*ICD-10* code H401), (2) had a visual field test code (E6991),^20^ (3) was prescribed IOP-lowering medications (α-agonists, β-blockers, carbonic anhydrase inhibitors, prostaglandin analogues, and combined medications),^29,30^ (4) received first diagnosis after January 1, 2004, and (5) no diagnoses of glaucoma secondary to eye inflammation (*ICD-10* code H404). For identification of patients with POAG, only those prescribed IOP-lowering eye drops more than twice, as in the incident RA cohort, were enrolled. The time of event was defined as the date of outpatient visit fulfilling the above definition.

### Risk-Set Matching

Although the cohort was constructed retrospectively, this study was able to address many of the limitations inherent in a retrospective design through risk-set matching with time-dependent propensity score.^31^ First, association between RA and risk of developing POAG was observed with time-dependent propensity score matching to adjust for confounders.^32^ As with propensity score, we estimated hazard components from the Cox proportional hazard model, with January 1, 2004, as the baseline and RA as the event.^32^ All variables in Table 1, collected during the 2 years before baseline (2002-2003), were included as covariates. Specifically, age was included as a continuous variable, whereas sex, household income level (decile), residential district (rural and urban), disability, Charlson comorbidity Index score, number of hospital admissions and outpatient visits, and medical history were included as categorical variables. The Charlson Comorbidity Index was calculated by weighting and scoring of comorbidities, with additional points for comorbidities associated with health outcomes of patients.^33,34^ Prescription for greater than 90 days of antihypertensive, antidiabetic, and lipid-lowering agents was considered important for patients who had been prescribed medications for preexisting conditions.

**Table 1.** Baseline Characteristics of Patients With Rheumatoid Arthritis and Their Risk Set-Matched Cohort^a^

Characteristic	Patients, No. (%)		Standardized difference^b^
	Rheumatoid arthritis cohort (n = 2049)	Matched cohort (n = 8196)	
Sex			
Men	551 (26.9)	2204 (26.9)	0
Women	1498 (73.1)	5992 (73.1)	
Age, mean (SD), y	67.70 (4.84)	67.70 (4.84)	0
Household income level			
Medical aid program	168 (8.2)	740 (9.0)	0.009
National health insurance premium decile			
First	183 (8.9)	788 (9.6)	
Second	97 (4.7)	348 (4.2)	
Third	126 (6.1)	466 (5.7)	
Fourth	118 (5.8)	478 (5.8)	
Fifth	140 (6.8)	511 (6.2)	
Sixth	141 (6.9)	584 (7.1)	
Seventh	199 (9.7)	865 (10.6)	
Eighth	227 (11.1)	932 (11.4)	
Ninth	279 (13.6)	1070 (13.1)	
Tenth	371 (18.1)	1414 (17.3)	
Residential district			
Urban^c^	826 (40.3)	3255 (39.7)	0.01
Rural^d^	1223 (59.7)	4941 (60.3)	
Registered disability			
No	2041 (99.6)	8163 (99.6)	0.002
Yes	8 (0.4)	33 (0.4)	
Charlson Comorbidity Index			
0	875 (42.7)	3600 (43.9)	0.05
1	613 (29.9)	2503 (30.5)	
2	361 (17.6)	1377 (16.8)	
3	125 (6.1)	444 (5.4)	
≥4	75 (3.7)	272 (3.3)	
Outpatient visits, No.			
0	164 (8.0)	707 (8.6)	0.05
1-9	287 (14.0)	1248 (15.2)	
10-19	364 (17.8)	1514 (18.5)	
20-29	457 (22.3)	1823 (22.2)	
30-39	336 (16.4)	1242 (15.2)	
≥40	441 (21.5)	1662 (20.3)	
Hospital admissions, No.			
0	1579 (77.1)	6513 (79.5)	0.06
1	305 (14.9)	1087 (13.3)	
≥2	165 (8.1)	596 (7.3)	
Antidiabetic agents^e^	168 (8.2)	559 (6.8)	0.05
Antihypertensive agents^f^	665 (32.5)	2398 (29.3)	0.07
Lipid-lowering agents^g^	121 (5.9)	384 (4.7)	0.05
Chronic kidney disease	5 (0.2)	17 (0.2)	0.008
Stroke	49 (2.4)	127 (1.5)	0.06
Malignant neoplasm	52 (2.5)	191 (2.3)	0.01

^a^ At the incidence date of rheumatoid arthritis in each patient, controls were matched according to the propensity score estimated by the Cox proportional hazards model.

^b^ Standardized difference of less than 0.1 (10%) is generally considered negligible.

^c^ Urban areas include Seoul, Busan, Daegu, Incheon, Gwangju, Daejeon, and Ulsan, which are larger than metropolitan cities.

^d^ Rural areas include Gyeonggi-do, Gangwon-do, Chungcheongbuk-do, Chungcheongnam-do, Jeollabuk-do, Jeollanam-do, Gyeongsangbuk-do, Gyeongsangnam-do, Jeju-do, and Sejong-si.

^e^ Antidiabetic agents include α-glucosidase inhibitors, biguanides, dipeptidyl peptidase-4 inhibitors, glucagon-like peptide-1 receptor agonists, insulin, meglitinide, sulfonylureas, and thiazolidinediones.

^f^ Antihypertensive agents include angiotensin-converting enzyme inhibitors, angiotensin II receptor blockers, β-blockers, calcium channel blockers, and diuretics.

^g^ Lipid-lowering agents include statins, fibrates, nicotinic acid, and ezetimibe.

Second, to address the limitations of retrospective studies, when a patient with RA was first identified, individuals at risk of RA but not yet at onset were matched among patients of the same age and sex as the identified patient with RA. Thereafter, for matched controls, diagnosis date of the matched patient with RA was set as the index date (time zero), and progress was observed. We repeated this risk-set matching method for all subsequent patients with RA.^35,36^

Finally, 1:4 matching on propensity score was successively performed for each risk-set with a nearest neighbor matching algorithm with a maximum caliper width of 0.1 for the hazard components.^37^ To make the matching independent of future events, matched control patients (control group) were set to be either those who never developed RA or those who had not yet developed RA. In other words, a patient with RA in the incident RA cohort was included in this study as either a patient with RA or as a matched control for another patient with RA whose time zero (date of incidence) was prior to theirs.^38^ We then removed matched patients from the following risk set to generate nonoverlapping samples. The same process was repeated within consecutive risk sets until patients with RA were no longer represented in these sets.

### Statistical Analyses

We used standardized difference to compare the distribution of baseline characteristics between RA and matched control cohorts. A standardized difference less than 10% (0.1) is generally considered a negligible association.^39,40^ Kaplan-Meier survival curves were used to observe the cumulative incidence curves of POAG and a stratified log-rank test was conducted to compare Kaplan-Meier curves of the matched cohort.^41^ Cumulative incidence of POAG and 95% CI were calculated by the product limit (Kaplan-Meier) method of survival probability. We calculated incidence rate (IR) of POAG and 95% CI with a generalized estimating equation using a Poisson distribution and expressed as number of POAG cases per 100 000 person-years. The association between RA and POAG was expressed as a hazard ratio (HR) using the Cox proportional hazards model with a robust variance estimator that accounts for clustering within matched pairs.^41^ For each patient with RA and their matched control, the date of RA incidence was set as the time zero (index date), the starting point for survival analysis. We defined survival time (POAG-free time) as months from time zero to the time of POAG occurrence, date of death, or December 31, 2013, whichever came first.^42^ Proportional hazard assumption was assessed by a Cox model with log transformation of the negative log of estimated survivor function, Schoenfeld residuals, cumulative sums of martingale residuals, and a supremum test. A Fine and Gray subdistribution hazard model was conducted along with cause-specific hazard model. Furthermore, subgroup analysis was performed according to 3 follow-up time frames (0-24, 0-48, and ≥49 months), age group, and sex. To exclude the use of corticosteroids as a potential confounder,^43^ a sensitivity analysis excluding corticosteroid users was performed, and to assess potential residual confounding such as frequent eye examinations in patients with RA, we also evaluated the risk of conjunctival and eyelid disorders during the follow-up period as negative control outcomes (eAppendix in the Supplement).^44,45^ A 2–sided *P* < .05 was considered significant. We performed all analyses using SAS statistical software version 9.4 (SAS Institute) and R statistical software version 4.0.3 (R Project for Statistical Computing). Data analysis was performed from November 2020 to July 2021.

## Results

**Figure 1.**
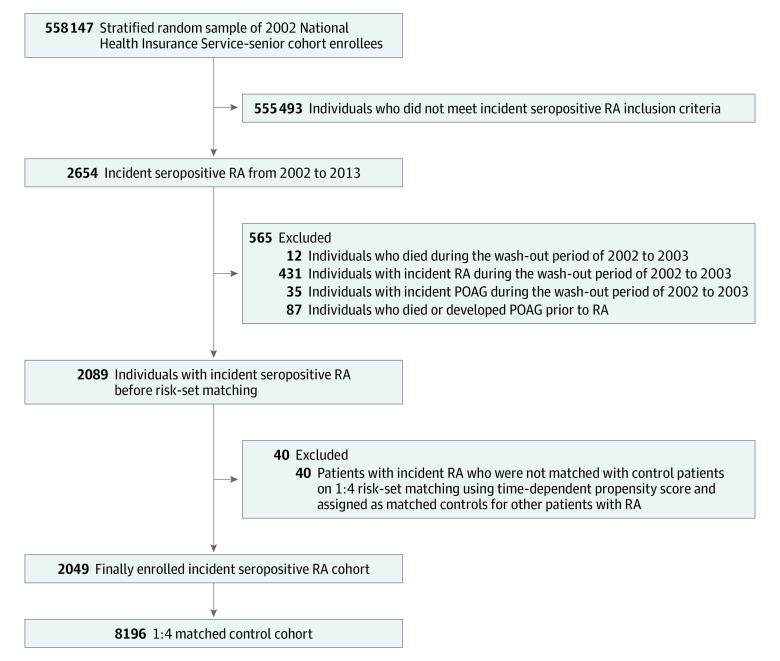
Participant Enrollment Flowchart POAG indicates primary open-angle glaucoma; RA, rheumatoid arthritis.

In our primary analysis, 2049 patients were incident seropositive for RA, and 8196 matched controls were assigned. The excluded patients and the reasons for exclusion are shown in Figure 1. There were 7490 women (73.1% of the cohorts), and the mean (SD) age of the participants was 67.70 (4.84) years. The distribution between the RA and the matched cohort was similar with the highest standardized difference of 6.9% in use of antihypertensive agents, and there was no significant difference between the 2 cohorts as the standardized difference of all covariates was less than 10% (Table 1). The mean (SD) follow-up period was 4.5 years (2.49), generating 46 142 person-years. During the follow-up period, 340 patients with POAG were identified.

**Figure 2.**
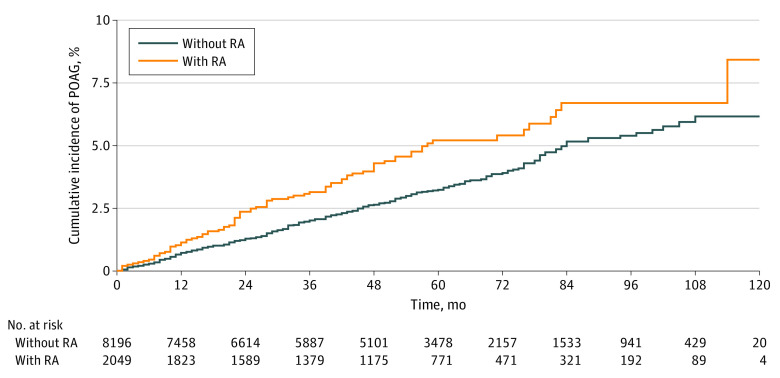
Cumulative Incidence of Primary Open-Angle Glaucoma (POAG) Among Patients With Incident Seropositive Rheumatoid Arthritis (RA) and Their Risk Set-Matched Controls During Follow-up *P* = .003 for stratified log-rank test.

The cumulative incidence of POAG was higher in the RA cohort than in the matched cohort during the entire follow-up period (Figure 2). The 2-year cumulative incidence risk of POAG was 2.36% in the RA cohort and 1.28% in the matched-control cohort; the 4-year cumulative incidence risk was 4.29% in the RA cohort and 2.64% in the matched control cohort (Table 2). During the entire follow-up period, 86 of 2049 seropositive patients with RA developed POAG for 8759 person-years (IR, 981.8 cases per 100 000 person-years; 95% CI, 794.3-1213.7 cases per 100 000 person-years), and in the matched cohort, 254 of 8196 individuals developed POAG for 37 383 person-years (IR, 679.5 cases per 100 000 person-years; 95% CI, 600.8-768.3 cases per 100 000 person-years). This difference implies that incident patients with RA were 1.44 times more likely to develop POAG than their matched controls (HR, 1.44; 95% CI, 1.13-1.84) (Table 3). The results using the Fine and Gray subdistribution hazard models and the cause-specific model were similar.

**Table 2.** Cumulative Incidence of Primary Open-Angle Glaucoma During Different Cumulative Time Frames

Cumulative time frame, y	Cumulative incidence, % (95% CI)^a^	
	Rheumatoid arthritis cohort	Matched cohort
1	1.13 (0.73-1.68)	0.72 (0.55-0.92)
2	2.36 (1.73-3.13)	1.28 (1.04-1.56)
3	3.14 (2.39-4.04)	2.01 (1.70-2.36)
4	4.29 (3.37-5.37)	2.64 (2.27-3.04)
5	5.21 (4.13-6.45)	3.23 (2.81-3.70)
6	5.40 (4.28-6.71)	3.90 (3.39-4.47)
7	6.70 (5.17-8.47)	5.16 (4.45-5.93)
8^b^	6.70 (5.17-8.47)	5.39 (4.64-6.21)
9^b^	6.70 (5.17-8.47)	6.16 (5.17-7.26)
10^c^	8.42 (5.17-12.67)	6.16 (5.17-7.26)

^a^ Cumulative incidence (%) was calculated by product limit (Kaplan-Meier) method of survival probability.

^b^ There was no development of primary open-angle glaucoma; between 84 and 113 months in rheumatoid arthritis cohort.

^c^ There was no development of primary open-angle glaucoma; between 109 and 120 months in matched cohort.

**Table 3.** Comparable Analysis of Rate of POAG for the Association of RA With Risk of POAG

Variables	Patients, No.	Cases of of POAG, No.	Person-years, No.	IR per 100 000 person-years (95% CI)	HR (95% CI)	*P* value
Full cohort						
Matched cohort	8196	254	37 383	679.5 (600.8-768.3)	1 [Reference]	.003
RA cohort	2049	86	8759	981.8 (794.3-1213.7)	1.44 (1.13-1.84)	
Time from diagnosis, mo						
0-24						
Matched cohort	8196	96	14 858	646.1 (531.0-786.2)	1 [Reference]	.001
RA cohort	2049	43	3644	1180.1 (875.6-1590.6)	1.83 (1.28-2.61)	
0-48						
Matched cohort	8196	177	26 557	666.5 (575.4-772.1)	1 [Reference]	<.001
RA cohort	2049	70	6388	1095.8 (867.1-1385.0)	1.65 (1.25-2.16)	
≥49						
Matched cohort	5035	77	10 826	711.2 (570.6-886.5)	1 [Reference]	.85
RA cohort	1157	16	2371	674.7 (413.3-1101.6)	0.95 (0.56-1.62)	
Sex						
Men						
Matched cohort	2204	61	9466	644.4 (501.3-828.2)	1 [Reference]	.07
RA cohort	551	22	2190	1004.6 (659.9-1529.2)	1.56 (0.96-2.54)	
Women						
Matched cohort	5992	193	27 917	691.3 (600.4-796.0)	1 [Reference]	.02
RA cohort	1498	64	6569	974.3 (762.1-1245.4)	1.41 (1.07-1.86)	
Age, y						
60-74						
Matched cohort	5796	198	28 968	683.5 (594.1-786.3)	1 [Reference]	.11
RA cohort	1449	59	6834	863.4 (668.5-1115.0)	1.26 (0.95-1.68)	
≥75						
Matched cohort	2400	56	8415	665.5 (515.2-859.5)	1 [Reference]	.001
RA cohort	600	27	1926	1402.2 (960.7-2046.7)	2.12 (1.34-3.35)	

Abbreviations: HR, hazard ratio; IR, incidence rate; POAG, primary open-angle glaucoma; RA, rheumatoid arthritis.

The increased risk of POAG in the RA cohort was 1.83-fold within 2 years after RA diagnosis (HR, 1.83; 95% CI, 1.28-2.61), and this trend persisted until 4 years after RA diagnosis (HR, 1.65; 95% CI, 1.25-2.16) (Table 3). Furthermore, among patients older than 75 years, the risk of POAG was 2.12 times higher in the RA cohort than in the matched controls (HR, 2.12; 95% CI, 1.34-3.35).

As a result of the sensitivity analysis to confirm the robustness of the results, the association between RA and risk of POAG was significant after excluding corticosteroid users (eTable 1 in the Supplement). The risk of conjunctival disorders (HR, 1.01; 95% CI, 0.83-1.25) and eyelid disorders (HR, 1.01; 95% CI, 0.80-1.29) as negative control outcomes showed no significant differences in the RA cohort vs matched controls (eTable 2 in the Supplement).

## Discussion

In this nationwide cohort study of older adults, we observed that incidence and risk of new-onset POAG in seropositive patients with RA were significantly higher than in matched controls during the 10-year follow-up period. To the best of our knowledge, this is the first longitudinal study to examine the association between RA and the subsequent risk of POAG using risk-set matching on propensity score, which addressed the limitations inherent to a retrospectively constructed cohort.^46^

Glaucoma exhibits a pathogenic pathway involving RGC loss, optic nerve damage, and corresponding visual field defects, but the underlying mechanisms of glaucomatous neuronal damage are not clear.^47^ Evidence that glaucoma is an autoimmune disease is steadily growing,^48^ including the discovery of an IgG autoantibody in human glaucomatous retina, and autoantibodies and T-cell responses to HSP detected in some patients with glaucoma.^49,50,51^ Increased serum autoantibodies against HSP27 observed in patients with glaucoma and IOP-independent RGC loss in animal models after immunization with HSP27 also provide evidence that the immune system is involved in the pathogenesis of glaucoma.^52^ Furthermore, recent data show that adaptive immune responses strongly exacerbate RGC loss in animal models of glaucoma.^53^

Recently published literature supported the hypothesis that RA and POAG may have common pathogenic or immunogenic components. The gene polymorphism of interleukin-10 is also one of the phenomena observed with equal frequency in RA and POAG.^54,55^ The use of drugs prescribed for patients with RA is being considered in animal models of glaucoma, to modulate neuroretina and optic nerve inflammatory response. This is part of the evidence that a paradigm shift is needed for glaucoma.^17,47^ Our findings support these theories from a clinical point of view, and the association of autoimmune disease with POAG should be investigated at least equivalently to diabetes or hypertension, whose associations with POAG are controversial.^56,57^

It is not likely that RA directly causes the development of POAG. In this study, in patients with new diagnoses of RA, the risk of POAG was increased more than 1.5-fold compared with patients without RA within 4 years of diagnosis, a fairly short time. Our results agree with those of previous studies,^58^ which confirmed an association between the diagnosis of specific autoimmune diseases including RA and subsequent dementia within 5 years. Considering that POAG has an insidious onset over decades, our results suggest that the immune complex involved in RA has the potential to simultaneously cause damage to tissues that are associated with the development of POAG including the retina or optic nerve. Specifically, various anti-HSP antibodies expressed in patients with RA are associated with the progressive degeneration of RGC, a characteristic of POAG,^59^ and T cell–mediated responses involved in the initiation of RA may be simultaneously involved in the onset of POAG.^50^ This is an important hypothesis to be confirmed in future studies with animal experiments or pathophysiological research.

Moreover, the association between RA and risk of POAG was evident in the older sector of our group. It is well known that the prevalence of associated systemic symptoms, progression of the disease, and functional outcomes may vary depending on the age of onset of RA.^60^ It is assumed that distinct characteristics of laboratory findings or phenotypes in late-onset RA, which are different from those of younger-onset RA,^61^ are associated with RA and subsequent risk of POAG, but additional studies should be considered.

### Strengths and Limitations

This study has several strengths. First, we investigated the association between RA and risk of POAG with risk-set matching using a nationwide cohort based on insurance claims, which is currently one of the best available methods. Risk-set matching enables analyses similar to randomized experiments in observational studies where randomized clinical trials cannot be implemented, such as in our study, and this method is one of the important ways to reduce the immortal time bias in epidemiological studies.^62,63^ We conducted the analysis considering that risk-set matching is a method to emulate the design and intention-to-treat analysis of randomized clinical trials. Thus, when a patient with RA was identified, patients at risk were matched by the propensity score, and changes of covariates after time zero were not considered to prevent immortal time bias. However, we assessed the robustness of our findings via sensitivity analysis, subgroup analysis, and use of negative control outcomes. Second, the NHIS-Senior cohort used in this study is a large sample with fairly low follow-up loss over a 12-year period because of the nature of national administrative claim data. Third, the NHIS-Senior cohort represents the entire sector of the Korean population aged 60 years and older. As the NHIS-Senior cohort was not limited to a particular hospital or organization, the study patients can be considered representative of the entire population, without bias. Therefore, this study is representative of patients with RA aged 60 years or older in Korea. Finally, this is the first study that we know of to report the association between RA and subsequent risk of POAG using risk-set matching with time-dependent propensity score, which mimics a prospective design to address the limitations of retrospective claim data. Furthermore, our results may provide improved awareness and evidence for a common mechanism regarding autoimmune components in development and progression of RA and POAG. Our findings also suggest that senior patients with RA should be closely monitored for subsequent development of POAG in clinical settings.

Our study has several inherent limitations. First, unhealthy behaviors, such as smoking history and obesity, which are factors known to be associated with the risk of RA or POAG, and severity or phenotypes of RA were not investigated in the NHIS-Senior cohort.^64^ Second, inaccurate diagnosis of RA and POAG obtained from insurance claim data was a possibility. However, to address these limitations, we defined RA and POAG by limiting the cases where specific prescription drugs (DMARD or IOP-lowering eye drops) and tests were also claimed, in addition to the *ICD-10* code. Third, causality cannot be inferred owing to the observational nature of the study, even though we adjusted for possible confounders and used specific statistical analyses to help emulate a prospective study design.

## Conclusions

In this risk-set matched cohort study, senior patients with RA had an increased risk of POAG compared with matched controls. The association between RA and subsequent risk of developing POAG was evident within 2 years after RA diagnosis, and in people aged 75 years or older. It is possible that POAG may have an autoimmune component such as RA. Further research is needed to investigate this and explore the underlying pathogenic mechanisms.

## Notes

Supplement. **eAppendix.** Supplemental Methods **eTable 1.** Sensitivity Analysis of the Risk of Development of Primary Open-Angle Glaucoma in Adults With Seropositive Rheumatoid Arthritis **eTable 2.** Hazard Ratio of the Primary and Negative Control Outcomes Following Diagnosis of Rheumatoid Arthritis vs Matched Controls
eReferences
