# Supplementary material for: Development of Open-Angle Glaucoma in Adults With Seropositive Rheumatoid Arthritis in Korea
Source: JAMA Netw Open. 2022 Mar 21;5(3):e223345. doi: 10.1001/jamanetworkopen.2022.3345 (PMC8938713; doi:10.1001/jamanetworkopen.2022.3345)
Supplement: Supplement. — eAppendix. Supplemental Methods eTable 1. Sensitivity Analysis of the Risk of Development of Primary Open-Angle Glaucoma in Adults With Seropositive Rheumatoid Arthritis eTable 2. Hazard Ratio of the Primary and Negative Control Outcomes Following Diagnosis of Rheumatoid Arthritis vs Matched Controls eReferences [file jamanetwopen-e223345-s001.pdf]

Supplemental Online Content

Kim SH, Jeong SH, Kim H, Park EC, Jang SY. Development of open-angle glaucoma in adults with seropositive rheumatoid arthritis in Korea. *JAMA Netw Open*. 2022;5(3):e223345. doi:10.1001/jamanetworkopen.2022.3345

eAppendix. Supplemental Methods

eTable 1. Sensitivity Analysis of the Risk of Development of Primary Open-Angle Glaucoma in Adults With Seropositive Rheumatoid Arthritis

eTable 2. Hazard Ratio of the Primary and Negative Control Outcomes Following Diagnosis of Rheumatoid Arthritis vs Matched Controls

eReferences

This supplemental material has been provided by the authors to give readers additional information about their work.

© 2022 Kim SH et al. *JAMA Network Open.*

eAppendix. Supplemental Methods

Sensitivity Analysis

We performed the sensitivity analysis excluding corticosteroid users to evaluate whether the use of corticosteroids acts as a potential confounder in the association between RA and risk of POAG, since the use of corticosteroids may increase IOP and increase the risk of POAG.

For the sensitivity analysis, we investigated the duration of corticosteroid use in NHIS-Senior cohort enrollees. Next, as the general population as well as incident RA patients are likely to use corticosteroids for various reasons, we created 5 cohorts excluding subjects with the following durations of corticosteroid use: 15 days or more, 1 month or more, 3 months or more, 6 months or more, 12 months or more. From each cohort, RA patients and their matched controls were extracted in the same method as main manuscript and Figure 1. After that, survival analysis were performed to evaluate the increased risk of POAG in RA patients, excluding corticosteroid effects.

Use of Negative Control Outcomes

To assess whether the difference in the risk of developing POAG between the RA cohort and their matched controls was the result of residual confounding from unmeasured covariates such as frequent eye exams in RA patients, we also measured the risk of conjunctival and eyelid disorders as negative control outcomes.

As many ocular manifestations (e.g., keratoconjunctivitis, dry eye, scleritis, episcleritis, uveitis, cataract, secondary glaucoma, retinal detachment) have been reported to be related to RA or autoimmune disease,[1-3] we selected conjunctival and eyelid disorders unrelated to inflammatory response including entropion, trichiasis, ectropion, pterygium, and conjunctival scars or degenerations (ICD-10 code: H01, H11). Risk-set matching was performed in the same

© 2022 Kim SH et al. *JAMA Network Open.*

method as main manuscript and Figure 1. Survival analyses was performed in the same methods as main manuscript to investigate the risk of negative control outcomes in RA patients vs matched controls.

© 2022 Kim SH et al. *JAMA Network Open.*

eTable 1. Sensitivity Analysis of the Risk of Development of Primary Open-Angle Glaucoma in Adults with Seropositive Rheumatoid Arthritis

Variables	Patients, No.	Cases of POAG, No.	Person-years	IR per 100 000 person-years (95% CI)	HR (95% CI)	*P* value
**Excluding patients who used steroids for more than 12 months**						
Matched cohort	6,660	216	30,002	720.0 (630.3 – 822.4)	1 [Reference]	<.001
RA cohort	1,665	80	6,943	1152.2 (924.8 – 1435.4)	1.61 (1.25 – 2.07)	
**Excluding patients who used steroids for more than 6 months**						
Matched cohort	6,100	212	27,489	771.2 (673.2 – 883.6)	1 [Reference]	.005
RA cohort	1,525	72	6,351	1133.6 (898.8 – 1429.7)	1.47 (1.12 – 1.91)	
**Excluding patients who used steroids for more than 3 months**						
Matched cohort	5,424	168	24,282	691.9 (595.7 – 803.6)	1 [Reference]	<.001
RA cohort	1,356	65	5,595	1161.8 (910.1 – 1483.1)	1.68 (1.26 – 2.23)	
**Excluding patients who used steroids for more than 1 month**						
Matched cohort	4,500	155	20,213	766.9 (654.1 – 899.0)	1 [Reference]	<.001
RA cohort	1,125	61	4,636	1315.7 (1022.5 – 1693.0)	1.71 (1.27 – 2.31)	
**Excluding patients who used steroids for more than 15 days**						
Matched cohort	3,860	139	17,183	808.9 (684.3 – 956.3)	1 [Reference]	.002
RA cohort	965	53	3,934	1347.4 (1027.7 – 1766.5)	1.66 (1.21 – 2.29)	

Abbreviations: POAG, primary open-angle glaucoma; IR, incidence rate; HR, hazard ratio; CI, confidence interval; RA, rheumatoid arthritis.

© 2022 Kim SH et al. *JAMA Network Open.*

eTable 2. Hazard Ratio of the Primary and Negative Control Outcomes Following Diagnosis of Rheumatoid Arthritis vs Matched Controls.

Outcomes	Patients, No.	Cases of outcome, No.	Person-years	IR per 100 000 person-years (95% CI)	HR (95% CI)	*P* value
**Primary outcome: POAG**						
Matched cohort	8,196	254	37,383	679.5 (600.8 – 768.3)	1 [Reference]	
RA cohort	2,049	86	8,759	981.8 (794.3 – 1213.7)	1.44 (1.13 – 1.84)	.003
**Negative control 1 (Disorders of conjunctiva): Pterygium, Conjunctival scars and degeneration**						
Matched cohort	7,792	471	34,878	1350.4 (1234.4 – 1477.4)	1 [Reference]	
RA cohort	1,948	114	8,276	1377.4 (1146.2 – 1655.2)	1.01 (0.83 – 1.25)	.86
**Negative control 2 (Disorders of eyelid): Entropion, Trichiasis, Ectropion**						
Matched cohort	8,000	333	36,345	916.2 (824.9 – 1017.7)	1 [Reference]	
RA cohort	2,000	81	8,713	929.6 (748.4 – 1154.7)	1.01 (0.80 – 1.29)	.90

Abbreviations: IR, incidence rate; HR, hazard ratio; CI, confidence interval; POAG, primary open-angle glaucoma; RA, rheumatoid arthritis.

© 2022 Kim SH et al. *JAMA Network Open.*

eReferences

1. Fujita M, Igarashi T, Kurai T, Sakane M, Yoshino S, Takahashi H. Correlation between dry eye and rheumatoid arthritis activity. *Am J Ophthalmol*. 2005;140(5):808-813.

2. Patel SJ, Lundy DC. Ocular manifestations of autoimmune disease. *Am Fam Physician*. 2002;66(6):991-998.

3. Black RJ, Hill CL, Lester S, Dixon WG. The association between systemic glucocorticoid use and the risk of cataract and glaucoma in patients with rheumatoid arthritis: a systematic review and meta-analysis. *PloS One*. 2016;11(11):e0166468.

© 2022 Kim SH et al. *JAMA Network Open.*
